# Temperature-dependent optical properties of some mixtures nematic liquid crystal

**DOI:** 10.1038/s41598-022-16750-x

**Published:** 2022-07-25

**Authors:** Zhila Alipanah, Mohammad Sadegh Zakerhamidi, Amid Ranjkesh

**Affiliations:** 1 Faculty of Physics, University of Tabriz, Tabriz, Iran; 2 Photonics Center of Excellence, University of Tabriz, Tabriz, Iran; 3 Condensed Matter Department, J. Stefan Institute, Jamova 39, Ljubljana, Slovenia

**Keywords:** Optics and photonics, Liquid crystals

## Abstract

The presence of optical anisotropy in liquid crystals (LCs) has caused these materials to have dual refractive indices: ordinary (n_o_) and extra-ordinary (n_e_). Many fundamental information about LCs can be found by looking at these refractive indices. In this work, the refractive indices of four mixtures nematic liquid crystal (NLC) have been studied as a function of temperature, and the relevant functions were then calculated. Subsequently, the order parameter of mentioned LCs was determined using three methods: Vuks, Haller, and the effective geometry parameter method. It was concluded that the obtained values are not significantly different and exhibit the same temperature dependence. The obtained results were evaluated in relation to the approach utilized.

## Introduction

The last few decades have seen an enormous growth in the development of novel devices based on optics and photonics technologies^1–4^. Therefore, materials and designs are constantly required in emerging modern technologies to build compact, weightless, highly adaptable, and effective optical devices^5–7^. Liquid crystals (LCs) are one of the most well-known materials in this field^8^. LCs, an intermediate phase of matter between isotropic fluid and solid crystal, combine the optical anisotropy of crystals with the molecular mobility of liquids, which is a crucial property of these systems^9,10^. The operation of LCs is confined to the liquid crystalline state—within a specific temperature range^11–13^. Meanwhile, the great sensitivity of their anisotropic features to temperature has led to the development of LCs that operates over a wide temperature range^14,15^. This has sparked a lot of interest in eutectic LC mixtures, due to the wide LC application temperature range and controllable optical and electro-optical properties in these application regions^15–17^. Because of having two refractive indices; ordinary n_o_ and extraordinary n_e_,—govern the application context of LCs; thus investigation on the optical anisotropy of LCs is increasingly important^9,10,18,19^. Studies on the optical anisotropy of LCs help to find out the relationship between macroscopic and microscopic properties in the LCs.

The order parameter is one of the most important parameters in the characterization of LCs, which can be determined in both microscopic and macroscopic forms. This parameter controls all physical properties of LCs in addition to verifying their degree of intrinsic long-range order [^18–21^ (19–20, 20–21)]. There are various methods for determining the order parameter, with techniques based on optical anisotropy being the most popular due to their high accuracy^22,23^. Among these, the Vuks method is an extensively utilized method that concts the LC’s molecular polarizability to its macroscopic refractive indices and determines the order parameter at the microscopic level^24^. Haller’s model is another one that uses refractive indices data as bulk tensorial features to estimate the macroscopic order parameter^25^. Moreover, the other standard techniques based on refractive indices, can be used for the measurement of the effective geometry parameter (α_eg_) for determine the order parameter^26–28^. α_eg_, as a macroscopic parameter, describes how light travels through an LC compound. Due to symmetry breakdown at the phase transition in the LC phases, light can be diverted towards the director's faulty orientation. Therefore, α_eg_ defines the degree of light deflection. The arrangement of molecules in LC naturally affects how light is transmitted. The order parameter of many nematic liquid crystals was recently determined using mentioned above optical approaches^12,22,29–31^.

In this work, firstly the refractive indices of some mixtures NLC were measured and their temperature dependence was investigated. Then, related LCs’ order parameter is calculated by using three methods: Vuks, Haller, and effective geometry parameter equation, after advancing the appropriate calculations. All calculations were based on data acquired from refractive indices measurements. The obtained results were evaluated in relation to the approach utilized.

## Theoretical background

### Steps for calculating the order parameter

The dielectric constant (ε) of anisotropic media is related to molecular polarizability (α) and molecular packing density (N) according to the Clausius–Mossotti equation^32^:

1 $$ (\varepsilon - 1)/\left( {\varepsilon + 2} \right) = \left( {4\pi /3} \right)N\alpha $$

In the optical frequencies, it can be substituted ε with n^2^ and obtain the Lorentz-Lorentz equation^32^:

2 $$ (n^{2} - 1)/\left( {n^{2} + 2} \right) = \left( {4\pi /3} \right)N\alpha $$

Due to the anisotropic nature of LCs, these materials have two refractive indices, which Vuks proposed a semi-empirical equation that correlates microscopic molecular polarizabilities to macroscopic refractive indices for anisotropic medium considering an isotropic local field among various researches^16,24^:

3 $$ \left( {n_{e,o}^{2} - 1} \right)/\left( {n^{2} + 2} \right) = \left( {4\pi /3} \right)N\alpha_{e,o} $$

In this, α_e_ and α_o_ represent extraordinary and ordinary molecular polarizabilities, respectively, where < n^2^ > is the mean value of the square of the refractive index which is defined as^29^:

4 $$ n^{2} = \left( {n_{e}^{2} + 2n_{o}^{2} } \right)/3 $$

Also, the refractive indices of LC can be expresses as a function of < n > and birefringence (Δn = n_e_ − n_o_) using this approach^24,29^:

5 $$ {\text{n}}_{{\text{e}}} = { }\left\langle {\text{n}} \right\rangle + \left( {2/3} \right)\Delta {\text{n}} $$

6 $$ {\text{n}}_{{\text{o}}} = { }\left\langle {\text{n}} \right\rangle - \left( {1/3} \right)\Delta {\text{n}} $$

In the following, the linear temperature dependency of $<\mathrm{n}>$ can be determined by evaluating the variation of the temperature-dependent effective molecular density using the experimental data of n_e_ and n_o_^30,31^:

7 $$ \sqrt {\left\langle {\text{n}} \right\rangle } = {\text{A}} + {\text{BT}} $$

The values of A and B are determined by fitting a curve that depicts the temperature dependence of $\sqrt {\left\langle {\text{n}} \right\rangle }$. In the next step, the Haller’s approximation is used to characterize the temperature dependency of birefringence^20^:

8 $$ \Delta {\text{n}} = \left( {\Delta {\text{n}}} \right)_{{\text{o}}} \left( {1 - \left( {T/T_{C} } \right)} \right)^{{\upbeta }} $$

where $({\Delta \mathrm{n})}_{\mathrm{o}}$ is the LC birefringence in the crystalline state, β is the LC material's characteristic constant, and T_C_ is its clearing temperature. The values of ${(\Delta \mathrm{n})}_{\mathrm{o}}$ and β can be obtained by using the linear curve fitting approach. Subsequently, the temperature–dependent refractive indices of LCs can then be derived by inserting values of ∆n and $\sqrt{<\mathrm{n}>}$ in Eqs. (5) and (6), resulting in the modified four-parameter model^30,31^:

9 $$ {\text{n}}_{{\text{o}}} = {\text{A}} + {\text{BT}} - \frac{1}{3}(\Delta {\text{n}})_{{\text{o}}} \left( {1 - \left( {T/T_{C} } \right)} \right)^{{\upbeta }} $$

10 $$ {\text{n}}_{{\text{e}}} = {\text{A}} + {\text{BT}} + \frac{2}{3}(\Delta {\text{n}})_{{\text{o}}} \left( {1 - \left( {T/T_{C} } \right)} \right)^{{\upbeta }} $$

So, Eqs. (9) and (10) can be utilized to directly compute the temperature variation rate of n_e_ and n_o_^29,30^:

11 $$ dn_{e} /dT = {\text{B}} - \left( {2/3} \right)\left( {\beta \left( {\Delta n} \right)_{o} /T_{C} } \right)\left( {1 - \left( {T/T_{C} } \right)} \right)^{{{\upbeta } - 1}} $$

12 $$ dn_{o} /dT = {\text{B}} + \left( {1/3} \right)\left( {\beta \left( {\Delta n} \right)_{o} /T_{C} } \right)\left( {1 - \left( {T/T_{C} } \right)} \right)^{{{\upbeta } - 1}} $$

In addition, using the modified-four parameter model, the cross-over temperature (T_co_) can be calculated. In this regard, it should be noted that the intermediate stage has a cross-over temperature, which is the transition temperature when dn_o_/dT = 0. Using this condition, it is possible to write^22^:

13 $$ T_{CO} = \left[ {1 - \left( { - 3BT_{C} /\left( {\Delta n} \right)_{o} \beta } \right)^{{\frac{1}{\beta - 1}}} } \right]T_{C} $$

After completing the calculations above, the normalized polarizability can be derived using ${\mathrm{n}}_{\mathrm{e}}$ and ${\mathrm{n}}_{\mathrm{o}}$ values, as well as $<{\mathrm{n}}^{2}>$^29,30^:

14 $$ \alpha_{e} /\left\langle \alpha \right\rangle = \left( {n_{e}^{2} - 1} \right)/\left( {n^{2} - 1} \right) $$

15 $$ \alpha_{o} /\left\langle \alpha \right\rangle = \left( {n_{o}^{2} - 1} \right)/\left( {n^{2} - 1} \right) $$

where:

16 $$ \left\langle {\upalpha } \right\rangle = \left( {\alpha_{e} + 2\alpha_{o} } \right)/3 $$

is the molecular mean polarizability. The ratio of normalized polarizabilities can be expressed as:

17 $$ \alpha_{e} /\alpha_{o} = \left( {n_{e}^{2} - 1} \right)/\left( {n_{o}^{2} - 1} \right) $$

Then, three different optical methods for determining the order parameter based on the temperature dependence of the refractive indices are utilized in this work. The Vuks’ model was employed as the first approach, where the order parameter can be expressed as^24,30^:

18 $$ {\text{S}}_{{{\text{vuks}}}} \left( {\Delta {\upalpha }/\alpha } \right) = \left( {n_{e}^{2} - n_{o}^{2} } \right)/\left( {n^{2} - 1} \right) $$

where $\Delta {\upalpha }\left( { = {\upalpha }_{{\text{e}}} - {\upalpha }_{{\text{o}}} } \right)$ and ${\upalpha }$ are polarizability anisotropy and mean molecular polarizability, respectively. To determine $\frac{{\Delta {\upalpha }}}{{\upalpha }}$, the linear part of ${\text{ln}}\left[ {\frac{{3\left( {{\text{n}}_{{\text{e}}}^{2} - {\text{n}}_{{\text{o}}}^{2} } \right)}}{{{\text{n}}_{{\text{e}}}^{2} + 2{\text{n}}_{{\text{o}}}^{2} - 3}}} \right]$ against ln $\left( {1 - \frac{{\text{T}}}{{{\text{T}}_{{\text{C}}} }}} \right)$ must be drawn and extrapolated to T = 0 K. By assuming that $\frac{{\Delta {\upalpha }}}{{\upalpha }}$ remains constant at all temperatures and putting this value into Eq. (18) at various temperatures, the order parameter value is derived.

The Haller’s approximation method is used to define the order parameter in the second approach, which the birefringence is converted to be linearly proportional to the order parameter. By disregarding the internal field effect, it is possible to write^30,31^:

19 $$ {\text{S}}_{{{\text{Haller}}}} = \left( {1 - \left( {{\text{T}}/T_{C} } \right)} \right)^{{\upbeta }} $$

In the third approach, the order parameter is defined using the effective geometry parameter (α_eg_)^14,27^:

20 $$ {\upalpha }_{{{\text{eg}}}} = { }n_{o} /n_{e} $$

By dividing Eq. (6) by Eq. (5) and using Eqs. (8) and (19), the order parameter value is obtained as follows^14,27^:

21 $$ {\text{S}}_{{{\upalpha }_{{{\text{eg}}}} }} = \left( {3\sqrt {n^{2} } /\left( {\Delta n} \right)_{o} } \right)\left( {\left( {1 - \alpha_{eg} } \right)/\left( {1 + 2\alpha_{eg} } \right)} \right) $$

## Experimental section

### Materials

In this work, four mixtures NLC (Merck Ltd.) have been selected as significant components in a variety of applications based on LC. Materials used are: MAT-131957 (LC-I), MAT-131958 (LC-II), ZKC-5102-LA (LC-III), ML-0682 (LC-IV). A set of desired physical properties is required depending on the application, not all of them are present in any single compound. As a result, research on liquid crystal mixtures of various shapes and structures has always proven to be lucrative from both a technological and basic standpoint. Two of selected LCs have positive dielectric anisotropy (∆ε) and the other two have negative. It is noteworthy that LCs with ∆ε > 0 have a dominant application in liquid crystal display (LCD) manufacturing. LCs with ∆ε < 0 are used to improve performance of LCDs, which in addition is highly regarded in optoelectronic applications^33–35^.

All investigated mixtures NLC are listed in Table 1 with relevant physical characteristics.

**Table 1:** Material parameters of the studied LCs: phase transition temperature (T_C_), birefringence (Δn), and the dielectric anisotropy (Δε).

Liquid crystal	T_C_ (K)	Δn (589 nm, 298 K)	Δε (1.0 kHz, 298 K)
LC-I (MAT-131957)	352.65	0.1070	− 4
LC-II (MAT-131958)	353.15	0.1066	− 4
LC-III (ZKC-5102-LA)	349.95	0.076	8.2
LC-IV (ML-0682)	350.25	0.063	13.1

### Refractive index measurement

Abbe refractometer with an accuracy of ± 0.00004 and a measurement range of 1.2 to 1.74 was used to measuring the LCs’ refractive indices (Bellingham Stanley Abe 60ED). To improve the border line contrast, refractometer ocular was fitted with a polarizer sheet to block exceptional ray. Also, refractometer's temperature was maintained by circulating water in a water bath temperature controller. The temperature was determined by placing a thermometer near the sample and measuring it with a precision of 0.01 °C.

## Results and discussion

### Temperature dependence of refractive indices and related parameters

**Figure 1:**
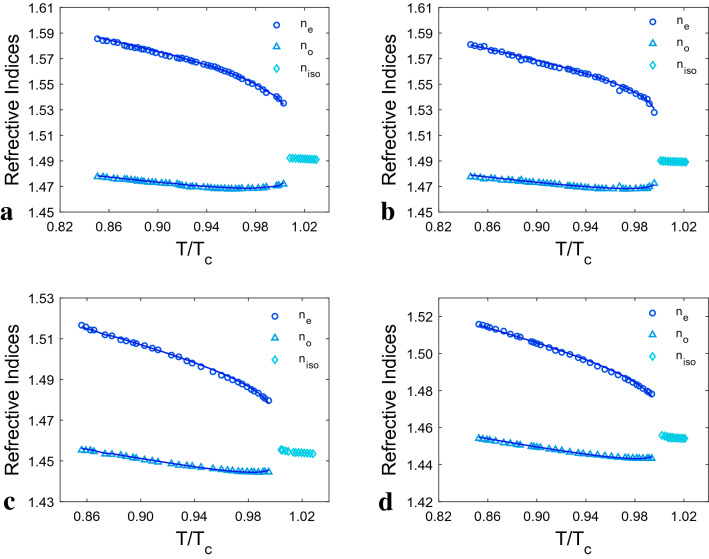
Temperature dependence of refractive indices: n_o_, n_e_ and n_iso_ (refractive index in isotropic state) for: (**a**) LC-I, (**b**) LC-II, (**c**) LC-III, (**d**) LC-IV. The experimental data are represented as symbols and the solid lines as individual fitting procedures.

Temperature dependences of the refractive indices are presented in Fig. 1. Symbols indicating experimental data and solid lines indicating fitting results using Eqs. (9) and (10). The birefringence for all studied LCs was calculated in room temperature using the measured refractive indices, which are roughly in conformity with the values provided in Table 1. All listed data in this table are extracted from LCs’ data sheets. This ensures that the refractometer is calibrated and that the measured data is accurate. The refractive indices of E7 were also measured, due to comparing our obtained results with a well-known LC^36–43^ which can be useful for analyzing the relevant results.

It is worth mentioning that the unknown constants in the modified four-parameter model, which are all LC characteristic constants, were calculated using the two-step fitting approach previously discussed in order to reach the fitting findings. Table 2 contains the related values. LC-I and LC-IV have the highest and lowest values for constants A and B, respectively. This trend was predictable from Eqs. (4) and (7), as well as the measured values of refractive indices, that LC with a higher mean value of the refractive index will have higher A and B values, even though the measurements were taken at the same temperature condition. In addition, according to Eq. (8), the LC with high $\Delta \mathrm{n}$ should have the highest value of ${(\Delta \mathrm{n})}_{\mathrm{o}}$ and the lowest value of β, respectively, which the values listed in Table 2 confirm this forecast. Also, when compared to the obtained results for E7 (see Table S1, supporting information), it can be seen that the values of A, B, and (∆n)_o_ belong to this LC more than the all studied LCs, although the corresponding value of β is less than that of all LCs.

**Table 2:** Fitting parameter for the mean refractive index and birefringence of the studied LCs and cross-over temperature (T_co_).

Liquid crystal	A	B	(Δn)_o_	_*β*_	T_co_ (K)
LC-I	1.637 ± 0.12%	− 0.000409 ± 0.12%	0.14025 ± 0.47%	0.1462 ± 0.47%	342.41 ± 0.05%
LC-II	1.635 ± 0.14%	− 0.000408 ± 0.14%	0.13643 ± 1.01%	0.1548 ± 1.01%	343.35 ± 0.05%
LC-III	1.597 ± 0.11%	− 0.000404 ± 0.11%	0.08539 ± 1.18%	0.1751 ± 1.18%	340.79 ± 0.05%
LC-IV	1.596 ± 0.10%	− 0.000403 ± 0.10%	0.08358 ± 0.37%	0.183 ± 0.37%	340.96 ± 0.05%

As shown in Fig. 1, for all LCs the extra-ordinary refractive index decline sharply as the temperature rises during the nematic phase, as the same as E7 (Fig. S1). The n_o_ decreases slowly at first and then increases as the temperature approaches the clearing temperature (T_C_). Due to the loss of anisotropy after the clearing temperature, n_o_ and n_e_ can no longer be described. As a result, this state is assigned a refractive index termed n_iso_, whose value decreases slowly as the temperature rises.

**Figure 2:**
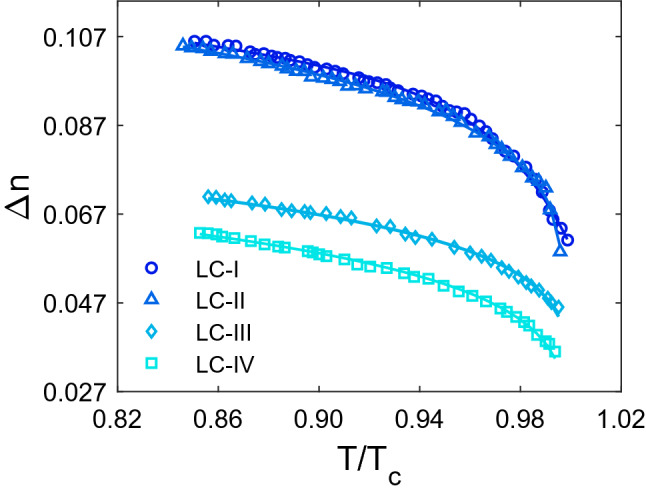
Temperature dependence of birefringence (Δn). The experimental data are represented as symbols and the solid lines as individual fitting procedures.

In the next step, the temperature dependence of birefringence ($\Delta \mathrm{n}$) was determined, as shown in Fig. 2. The highest and lowest $\Delta \mathrm{n}$ values are found in LC-I and LC-II, respectively. The $\Delta \mathrm{n}$ of all studied LCs decreases with increasing temperature throughout the nematic phase, which is based on the effect of temperature variations on intermolecular interactions^41,42^ between constituent molecules. Due to the loss of anisotropy, the $\Delta \mathrm{n}$ value approaches zero towards the clearing temperature. According to our results, during the nematic–isotropic phase transition, the refractive indices values change gradually as the temperature varies, but $\Delta \mathrm{n}$ values decline quickly as the temperature rises. It means that a small temperature fluctuation in the range of nematic–isotropic phase transition causes a large change in $\Delta \mathrm{n}$. By taking into account of the $\Delta \mathrm{n}$ values as shown in Fig. 2, a significant difference can be seen between LC-III and LC-IV compared with that in LC-I and LC-II. The optical anisotropy of the LCs, which is related to intrinsic features of these materials, is known to cause birefringence. Based on Eq. (8), dependence of ∆n on the structural parameters (β and (∆n)_o_) is evident. As mentioned earlier, a large value of (∆n)_o_ and a small value of β leads to a high amount of ∆n. According to Table 2, it can be seen that β value belong to LC-I and LC-II less than value belong to LC-III and LC-IV. Also, the value of (∆n)_o_ is high for LC-I and LC-II compared to other LCs. Thus, the high values of ∆n of LC-I and LC-II compared to LC-III and LC-IV can be predicted. Also, the difference between the values obtained for parameters β and (∆n)_o_ belonging to LC-III and LC-IV, in turn leads to a large difference between the birefringence of these materials.

Furthermore, in order to advance the comparison, E7’s birefringence was also measured, and its value was found to be higher than that of all the studied LCs. However, it exhibits the same temperature dependence as the birefringence of the all studied LCs investigated, as shown in Fig. S2 (supporting information).

**Figure 3:**
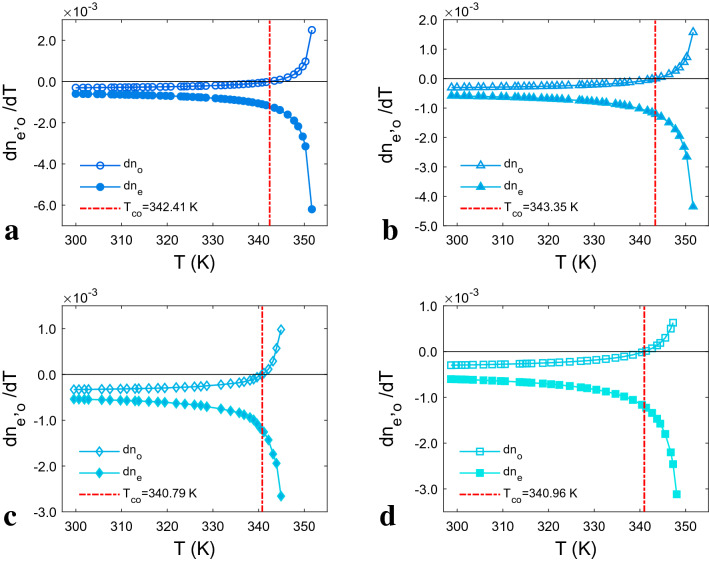
The temperature derivative of refractive indices (dn_o_/dT and dn_e_/dT) for: (**a**) LC-I, (**b**) LC-II, (**c**) LC-III, (**d**) LC-IV. The experimental data are displayed as symbols, and the solid lines obtained from the fitted curves.

Following that, the temperature gradient of ordinary (dn_o_/dT) and extra-ordinary (dn_e_/dT) refractive indices and cross-over temperature (T_CO_) of all studied LCs was determined. T_CO_ is an important criterion for determining the suitability of LC material for a specific application, particularly in display devices and nonlinear optics^43^. The calculations reveal that the dn_e_/dT for all LCs remains negative throughout their nematic range, (Fig. 3), whereas the dn_o_/dT values at the T_CO_ change sign as temperature rises. It means that the n_o_ decreases when temperature rises below T_CO_ and rises as temperature increases over T_C_. Up to T_CO_, the values of dn_o_/dT and dn_e_/dT progressively change, but once the operating temperature surpasses T_CO_, they change sharply. This is due to the LC's intermolecular arrangement being affected by temperature. Long-range orientational order (LRO) exists between molecules in the liquid crystalline phases. As the temperature approaches T_C_, LRO steadily decreases, laying the groundwork for the formation of short-range orientational order (SRO). The LRO reduces steadily until T_CO_, after which it drops abruptly due to the increase in molecular movement fluctuations with increasing temperature. In LC, the ability to have a high T_CO_ and a small defference bwetween T_CO_and T_C_ are critical. Because the lower T_CO_ value indicates that the material shows a high light deflection^43^. It is worth mentioning that the T_C_ effect is essential for reaching maximum T_CO_ values. The T_CO_ will be fairly high if the T_C_ of an LC material is significantly greater than the ambient temperature. Table 2 lists the T_CO_ values, with LC-II and LC-III exhibiting the maximum and minimum T_CO_, respectively, due to their highest and lowest T_C_. Our studied LCs have T_CO_ higher than E7. Also, the difference between T_CO_ and T_C_ is the smallest in LC-III compared to other ones.

**Figure 4:**
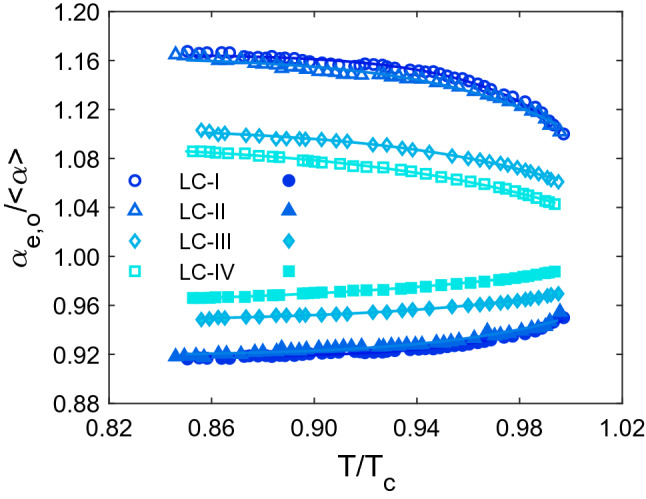
The variation of ordinary and extraordinary normalized polarizabilities with temperature. The experimental data are displayed as symbols, and the solid lines obtained from the fitted curves.

**Figure 5:**
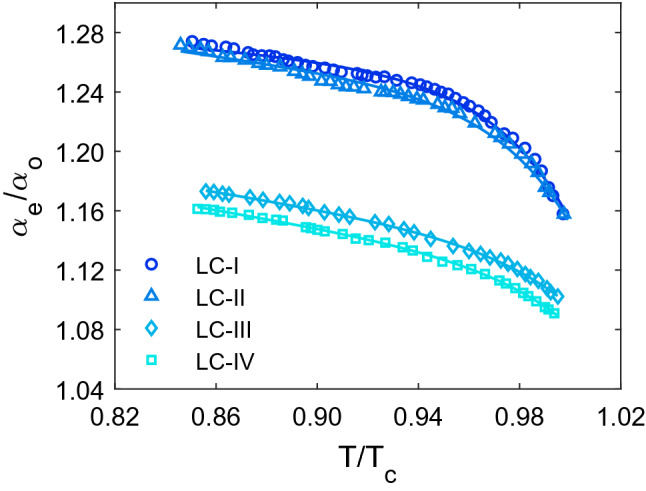
The variation of ratio of normalized polarizabilities (α_e_/α_o_) with temperature. The experimental data are represented as symbols and the solid lines as individual fitting procedures.

It is feasible to comprehend the analysis as accurately as possible by studying molecular polarizability. Therefore, normalized polarizabilities of ordinary (α_o_/ < α >) and extra-ordinary (α_e_/ < α >) components of all studied LCs, were directly determined using their refractive indices data. These calculations were carried out by putting the refractive indices values into Eqs. (14) and (15), and the result is shown in Fig. 4. As can be seen, the variations of α_o_/ < α > and α_e_/ < α > with temperature are comparable to those of the n_o_ and n_e_ which is seen in Fig. 1. As a result, as the temperature rises, the α_e_/ < α > and α_o_/ < α > values fall and increase, respectively. Because of the Lorentz-Lorentz equation (Eq. 2), which states a relationship between a molecule's polarizability and the refractive index of a substance made up of molecules with this polarizability. Hendrik Lorentz and the Danish scientist Ludwig Valentin Lorenz independently developed it in 1880 using macroscopic electrostatics^32^. Furthermore, as shown in Fig. 5, the temperature dependence of the ratio of normalized polarizability (α_e_/α_o_) of all studied LCs has the same performance as their birefringence temperature dependency. As the temperature rises, the ratio of normalized polarizabilities falls as the temperature rises, until it approaches unity around the T_C_ because the values for both α_e_ and α_o_ do not diverge in isotropic state. The results show that the LC-I and LC-IV have the highest and lowest normalized polarizability ratios, respectively. Thus, a minor change in refractive indices between the LC-I and LC-II results in a tiny difference in α_e_/α_o_ values for these two LCs. The shift in the value of the α_e_/α_o_ is mostly caused by temperature fluctuations in the n_o_ and n_e_ of LC, which alter the intermolecular interaction.

**Figure 6:**
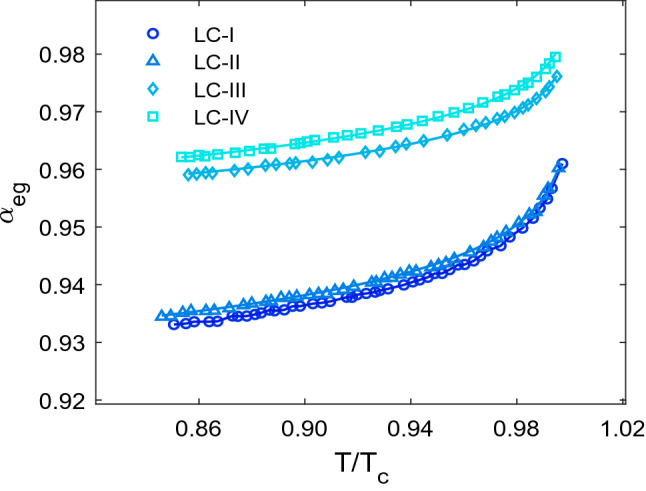
Effective geometry parameter (α_eg_) according to temperature. The experimental data are represented as symbols and the solid lines as individual fitting procedures.

To further the investigation, the effective geometry parameter of studied LCs, which is directly related to the LCs’ refractive indices values, was determined and the result of its temperature dependency is shown in Fig. 6. Because n_e_ is more temperature-sensitive than n_o_, α_eg_ rises with increasing temperature. When α_eg_ reaches unity, the equality of n_e_ and n_o_ in the isotropic phase, there is no longer any molecular orientational order in the LC, indicating that there is less light deflection. The LC-I has the lowest α_eg_ as compared to other studied LCs, according to statistics, because the difference between its n_e_ and n_o_ values is greater than the other LCs. Furthermore, the α_eg_ values of LC-I and LC-II are very similar. Because the difference between these LCs’ refractive indices is tiny.

### Order parameter

**Figure 7:**
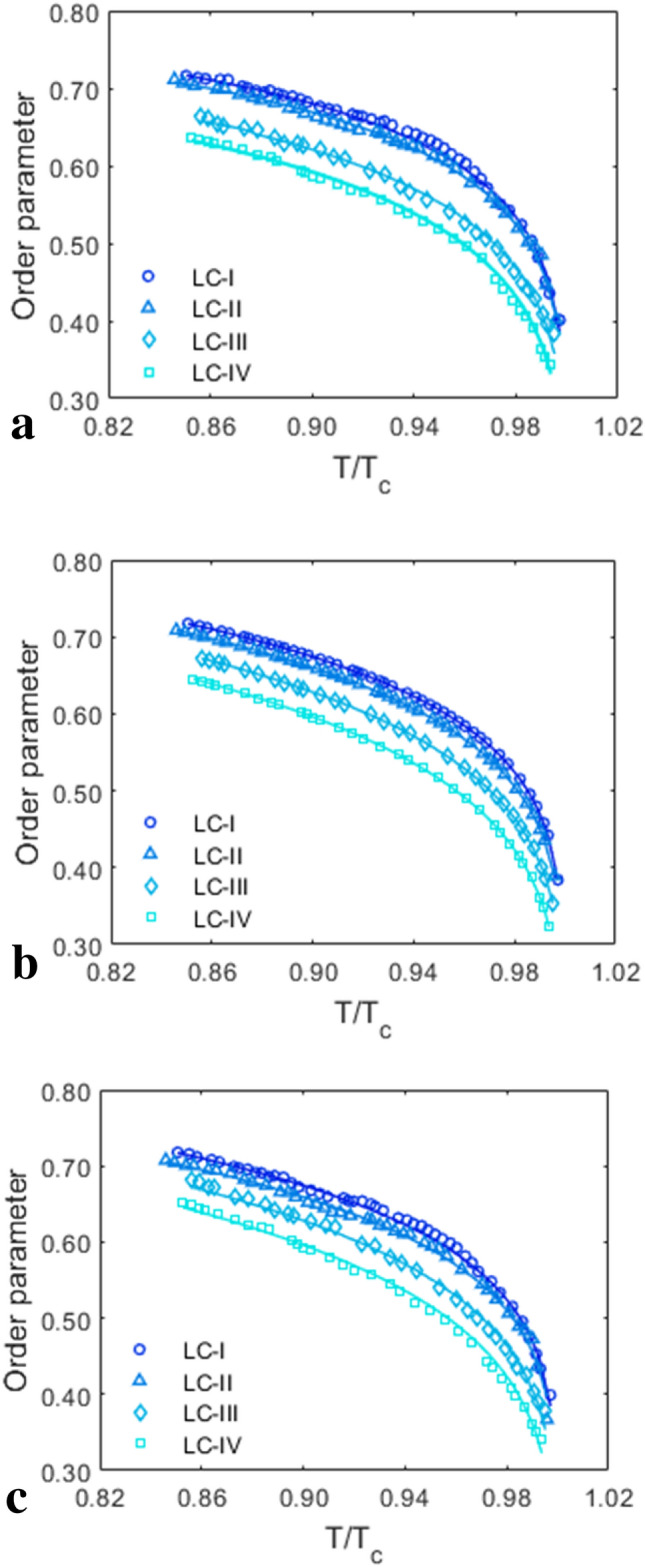
Temperature dependencies of order parameter obtained from different methods: (**a**) Vuks’ method, (**b**) Haller’s method and (**c**) effective geometry parameter technique. The experimental data are represented as symbols and the solid lines as individual fitting procedures.

The order parameter and its temperature dependence are extremely important for studying LCs’ orientational behavior. As previously mentioned, the order parameter of all studied LCs was determined by three different methods including Vuks, Haller, and effective geometry based on Eqs. (18), (19) and (20), respectively. Figure 7a shows obtained order parameters by using Vuks model. As shown, order parameter values of all studied LC compounds decline sharply with temperature across the whole nematic phase and then become zero in the isotropic state. This is because when the temperature rises, the order of the molecules' orientations decreases until they are completely disoriented in the isotropic state. As can be seen, the order parameter values of LC-I and LC-IV are greater and lower than the order parameter values of other studied LCs, respectively. Examining the procedure utilized can help to accurately investigate the rationale for the results. The scaling factor (∆α/α), as well as the refractive indices term $\left( {\frac{{{\text{n}}_{{\text{e}}}^{2} - {\text{n}}_{{\text{o}}}^{2} }}{{\left\langle {{\text{n}}^{2} } \right\rangle - 1}}} \right)$ based on Eq. (18) have an impact on the resulting values. A higher order parameter can be obtained on the lower ∆α/α with a greater value of $\frac{{\mathrm{n}}_{\mathrm{e}}^{2}-{\mathrm{n}}_{\mathrm{o}}^{2}}{<{\mathrm{n}}^{2}>-1}$. The scaling factors of LC-I, LC-II, LC-III, and LC-IV are 0.327, 0.328, 0.2199, and 0.227, respectively. In contrast, the values of $\frac{{\mathrm{n}}_{\mathrm{e}}^{2}-{\mathrm{n}}_{\mathrm{o}}^{2}}{<{\mathrm{n}}^{2}>-1}$ range from greater to less in LC-I, LC-II, LC-III, and LC-IV, as shown in Fig. S3 (supporting information), which represent the temperature dependency of $\frac{{\mathrm{n}}_{\mathrm{e}}^{2}-{\mathrm{n}}_{\mathrm{o}}^{2}}{<{\mathrm{n}}^{2}>-1}$. As a result, it is clear that $\frac{{\mathrm{n}}_{\mathrm{e}}^{2}-{\mathrm{n}}_{\mathrm{o}}^{2}}{<{\mathrm{n}}^{2}>-1}$ is the effective parameter in this model, the order of the order parameter is the same as $\frac{{\mathrm{n}}_{\mathrm{e}}^{2}-{\mathrm{n}}_{\mathrm{o}}^{2}}{<{\mathrm{n}}^{2}>-1}$ values, as determined by the order parameter values of the studied LCs.

Then, using Haller's technique, plotted in Fig. 7b, the same performance obtained by the Vuks’ method is observed. Using linear curve fitting approach on logarithmic of Eq. (8), the values of β is achieved. Our results show that LC-I with the high Δn value shows the lowest value of β amongst the other studied LCs. However, this result has already reported before^13,20^. Based on the model's equation, it can be deduced that β is the prominence parameter that determines the order parameter values. Therefore, the values listed in Table 2 can be used to sort the studied LCs’ order parameter values. Among them, LC-I and LC-IV have the greatest and lowest order parameter, respectively, due to having the lowest and highest values of β.

The order parameter determined using the effective geometry parameter model is then plotted against temperature in Fig. 7c. As can be seen, between the determined values the same behavior as previously indicated is observed. The temperature dependency of $\frac{1-{\mathrm{\alpha }}_{\mathrm{eg}}}{1+2{\mathrm{\alpha }}_{\mathrm{eg}}}$, a part of Eq. (21), is represented in Fig. S4 (supporting information), which the order of the obtained values for studied LCs is as follows: LC-I > LC-II > LC-III > LC-IV. Another part of Eq. (21), the temperature changes of $\frac{\sqrt{<{\mathrm{n}}^{2}>}}{{(\Delta \mathrm{n})}_{\mathrm{o}}}$ is presented in Fig. S5 (supporting information), which demonstrates that the order between the values is not as indicated above order. By combining the foregoing findings with the results shown in Fig. 7c, it can be concluded that using this approach, $\frac{1-{\mathrm{\alpha }}_{\mathrm{eg}}}{1+2{\mathrm{\alpha }}_{\mathrm{eg}}}$ is effective in identifying order parameter values obtained by geometry parameter model. As a result, the greatest and lowest order parameter values are found in LC-I and LC-IV respectively, with a very tiny difference in values between LC-I and LC-II.

It is also worth noting that the order parameter's values depending on the measurement method due to varying assumptions about the local field. As a result, the fluctuation of the order parameter with temperature for all three examined approaches was nearly equal, as shown in Fig. S4 (supporting information). The Vuks method is used to determine the microscopic order parameter, which is calculated using different quantities than the macroscopic one. Because the effect of the local field differs on different properties, the values calculated for the microscopic and macroscopic order parameters will differ. This microscopic order parameter is derived with refractive indices and molecular polarizability. While, Haller approach achieves macroscopic order parameter which structure parameters of LC are involved in the calculations. Also, effective geometry parameter model including the refractive indices in the relevant equation. All of the above approaches yield order parameter that temperature dependent. As a result, the main difference between the Vuks approach and the other two approaches is the simultaneous temperature dependence of molecular polarizability and refractive index, which results in a small and negligible difference in the values obtained when compared to the other mentioned two approaches at temperatures far from the T_C_ (see Fig. S6).

However, in order to progress the stated comparisons, it is worth noting that, according to Fig. S7 (supporting information), the order parameter belonging to E7 is larger than LC-III and LC-IV, and less than LC-I and LC-II, respectively, for all used techniques of calculation.

## Conclusion

We studied the temperature dependent refractive indices for several mixtures NLC. After calculating the birefringence and achieving the constants in the modified four-parameter model, the order parameter of the studied LCs was determined by following three methods: Vuks, Haller, and equation involving effective geometry parameter. The order parameter obtained using mentioned three approaches follows the same temperature dependence behavior. The order of the birefringence and order parameter values is the same in all LCs, confirming the refractive indices’ effective impact on the obtained values. The specified parameters’ values, from large to small, are assigned to LC-I, LC-II, LC-III, and LC-IV respectively. The relevant LC for the desired application can then be selected using these findings. When the T_CO_ of the studied LCs is examined, the T_CO_ pertaining to LC-II and LC-III are found to be the highest and lowest, respectively. The application temperature range of an LC with a modest difference between the T_C_ and T_CO_, is greater. As a result, among the studied LCs, LC-III has the highest applied temperature range as well as the least amount of light deflection. As a result, it could be advantageous in LCD manufacturing. Also, apart from the methods used, it can also be seen that LC-I has the greatest value of the order parameter among the studied LCs by looking at the values of the determined order parameters. Therefore, it could be an excellent fit for applications that are sensitive to order parameter and require a lot of contrast.

## Supplementary Information

Supplementary Information.

## Data Availability

The datasets used and/or analyzed during the current study available from the corresponding author on reasonable request. Supplementary Information is available from the Nature Publishing Online Library or from the authors.
