# Supplementary material for: Temperature-dependent optical properties of some mixtures nematic liquid crystal
Source: Sci Rep. 2022 Jul 25;12:12676. doi: 10.1038/s41598-022-16750-x (PMC9314397; doi:10.1038/s41598-022-16750-x)
Supplement: Supplementary file 1 — Supplementary Information. [file 41598_2022_16750_MOESM1_ESM.pdf]

Supplementary Information

Temperature-dependent optical properties of Some Mixtures Nematic Liquid Crystal

Zhila Alipanah [a*], Mohammad Sadegh Zakerhamidi[a,b], Amid Ranjkesh [c]

[a] *Faculty of Physics, University of Tabriz, Tabriz, Iran*

[b] *Photonics Center of Excellence, University of Tabriz, Tabriz, Iran*

[c] *Condensed Matter Department, J. Stefan Institute, Jamova 39, Ljubljana, Slovenia*

* Corresponding Author

Zhila Alipanah

Tel: +98–4133393353

Fax: +98–4133356030

E-mail: zh.alipanah@tabrizu.ac.ir and zh.alipanah@gmail.com

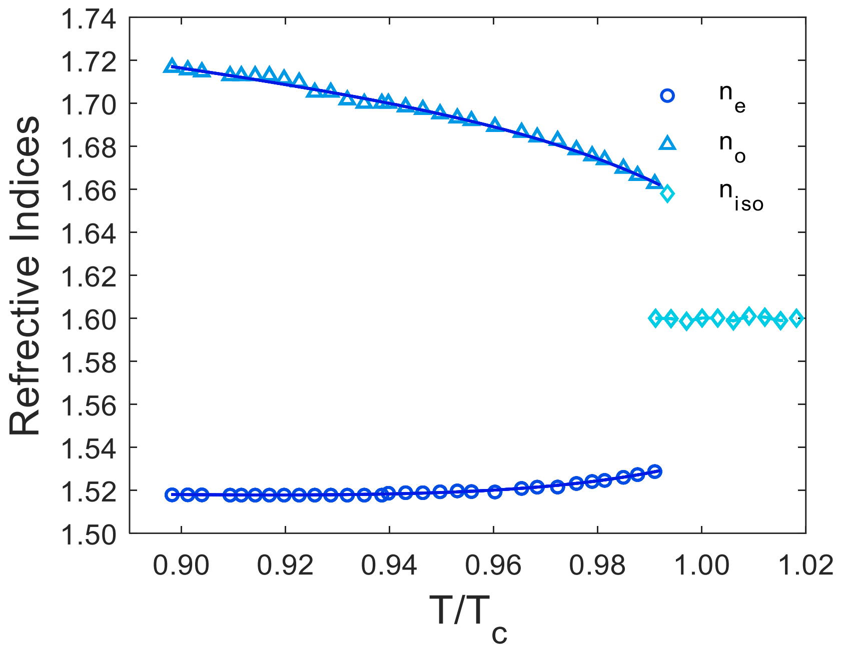

Fig. S1: (Color online) Temperature dependence of E7's refractive indices: n_o, n_e and n_{iso} (refractive index in isotropic state).

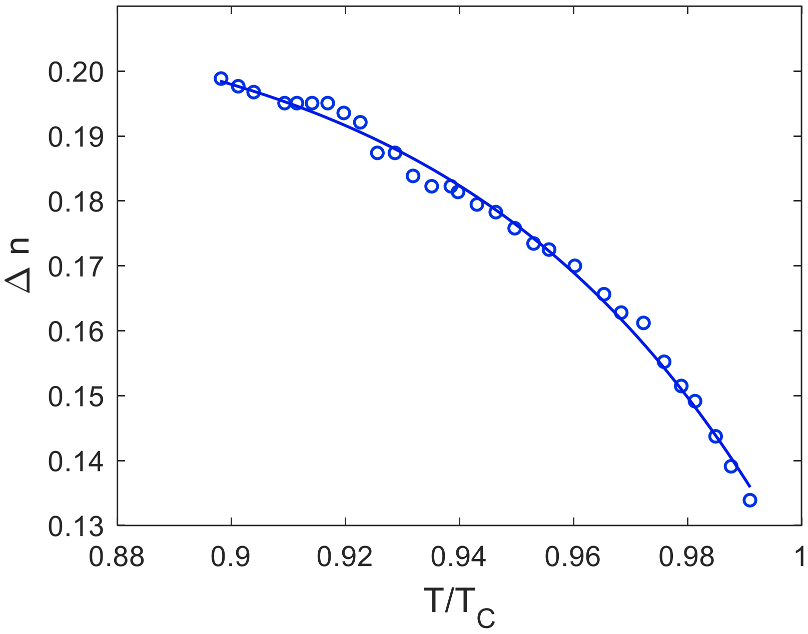

Fig. S2: (Color online) Temperature dependence of birefringence (Δn) of E7. The experimental data are represented as symbols and the solid lines as individual fitting procedures.

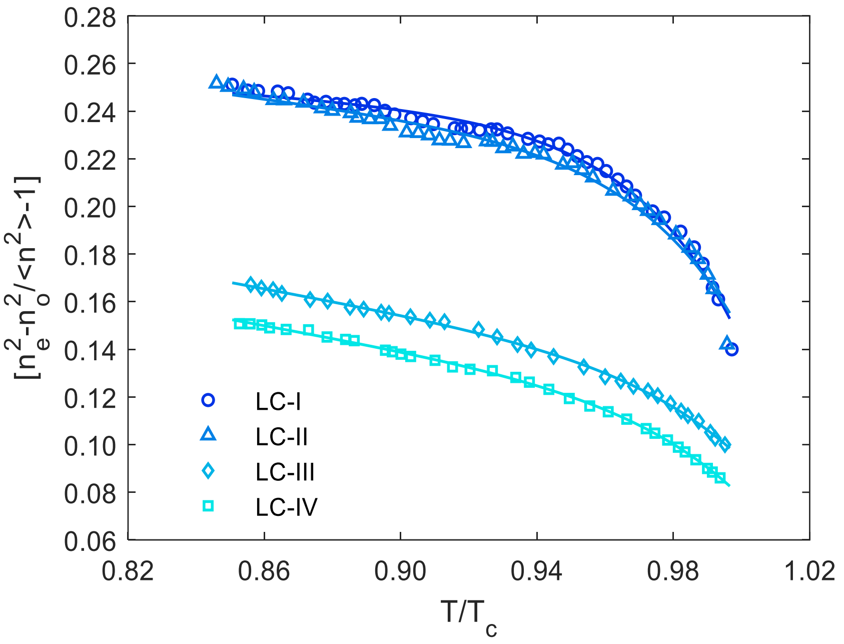

Fig. S3: (Color online) Temperature dependence of $\frac{n_e^2-n_o^2}{<n^2>-1}$ for the studied LCs using the Vuks' method. The experimental data are represented as symbols and the solid lines as individual fitting procedures.

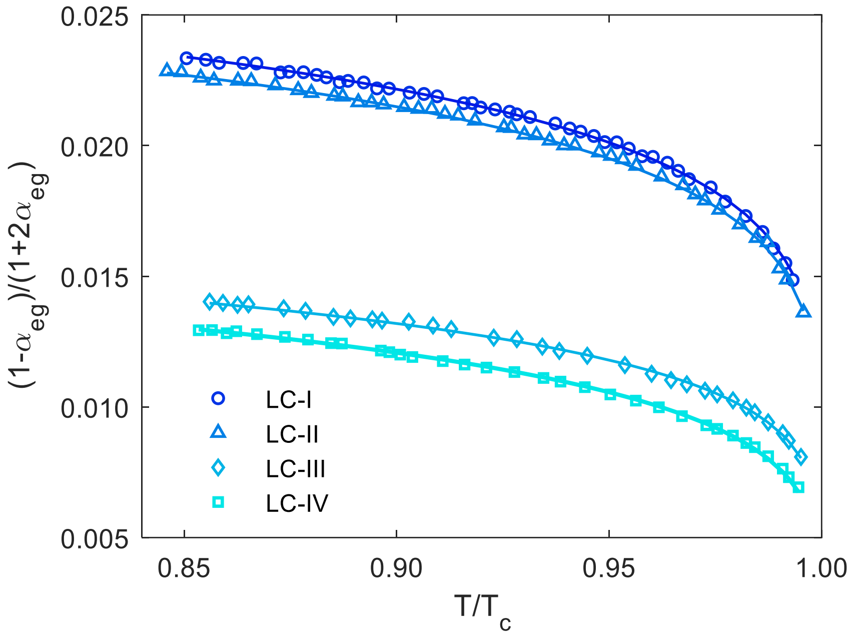

Fig. S4: (Color online) $\frac{1-\alpha_{eg}}{1+2\alpha_{eg}}$ according to temperature using effective geometry parameter technique. The experimental data are represented as symbols and the solid lines as individual fitting procedures.

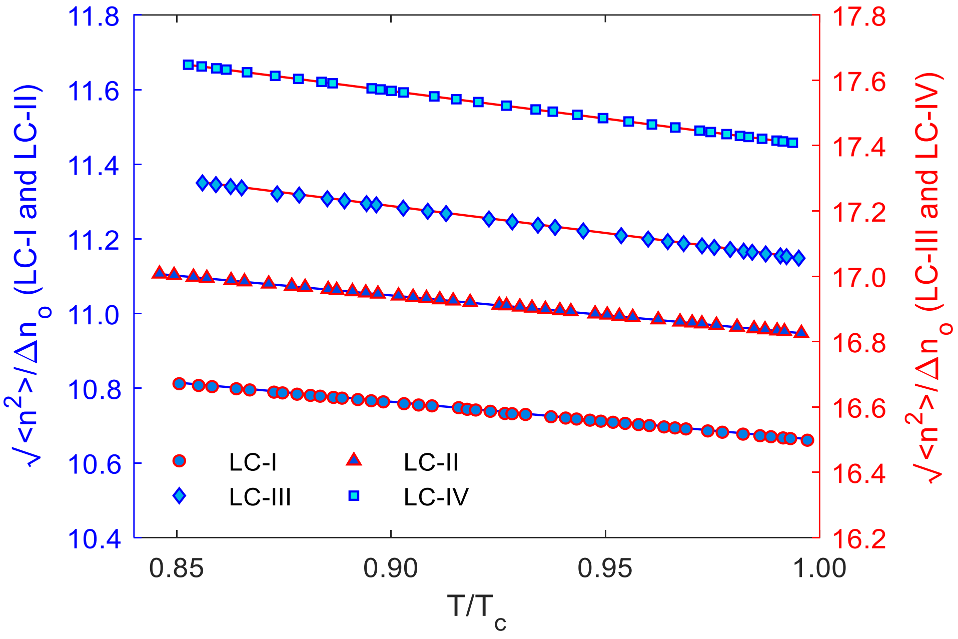

Fig. S5: (Color online) $\frac{\sqrt{<n^2>}}{(\Delta n)_o}$ according to temperature using effective geometry parameter technique.

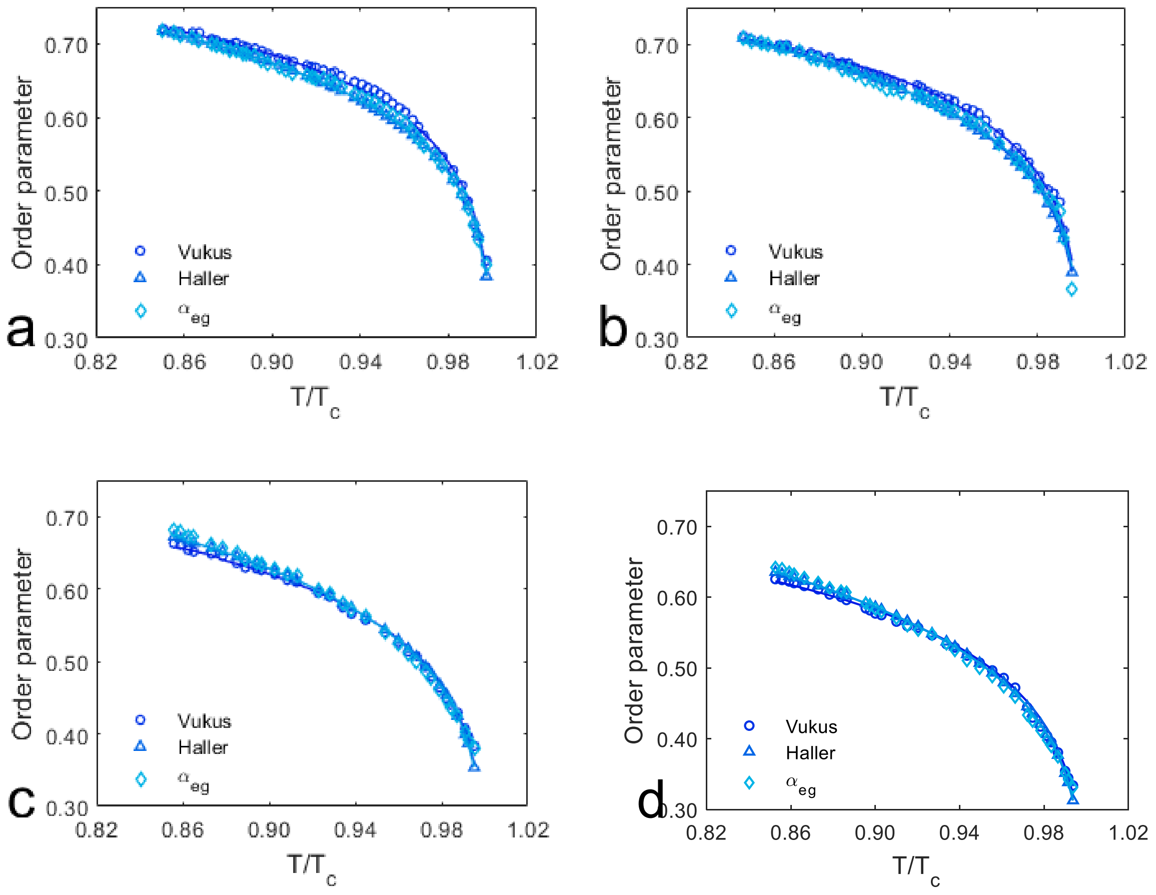

Fig. S6: (Color online) Temperature dependencies of order parameter obtained from different methods: Vuks' method, Haller's approximation method and effective geometry parameter for: a) LC-I, b) LC-II, c) LC-III, d) LC-IV.

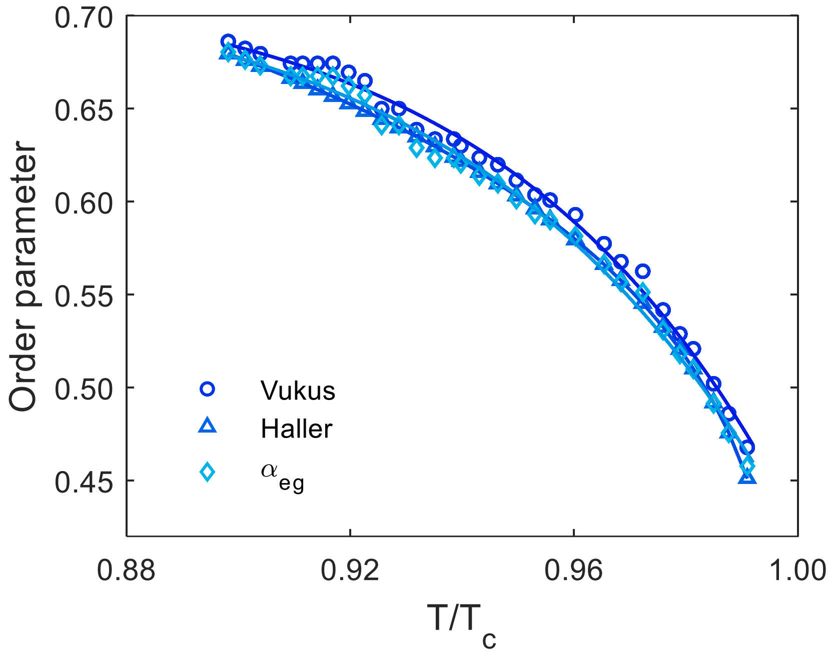

Fig. S7: (Color online) Temperature dependencies of order parameter obtained from different methods: Vuks method, Haller approximation method and effective geometry parameter equation for E7

Table. S1: Fitting parameter for the mean refractive index and birefringence of theE7 and cross-over temperature (T_{co})

**Liquid Crystal**	**A**	**B**	**$(\Delta n)_o$**	**β**	**T_{co} (K)**
E7	1.709 ±0.15%	-0.00041 ±0.15%	0.29275±0.37 %	0.1456±0.37 %	322.92±0.13%
